# Methods for Preserving Human Milk Cellular and Milk Fat Globule RNA

**DOI:** 10.1007/s10911-026-09601-2

**Published:** 2026-02-24

**Authors:** Zhengfei Li, Nicole Fischbein, Flora Jin, Wesley Mui, Raimundo Co, Amy Sarah Ginsburg, Victoria Laleau, Sarah Nyquist, Kimberly O O’Brien, Nadav Ahituv, Valerie Flaherman, Yarden Golan

**Affiliations:** 1 Division of Nutritional Sciences, Cornell University, Ithaca, NY USA; 2 Department of Pediatrics, University of California San Francisco, San Francisco, CA USA; 3 International Partnership for Human Development, Bissau, Guinea-Bissau; 4 Clinical Trials Center, University of Washington, Seattle, WA USA; 5 Gladstone Institutes, San Francisco, CA USA; 6 Department of Bioengineering and Therapeutic Sciences, University of California San Francisco, San Francisco, CA USA; 7 Institute for Human Genetics, University of California San Francisco, San Francisco, CA USA

**Keywords:** Human milk, RNA preservation, RNAlater, Milk fat globules, Milk cells, RNA integrity, Transcriptomics, Biobanking

## Abstract

Human breast milk contains RNA in various fractions, including milk cells and milk fat globules (MFG), making it a valuable resource for studying lactation physiology. However, preserving RNA integrity, especially in low-resource or at-home collection settings, is challenging due to rapid RNA degradation.

This study aimed to evaluate RNA preservation methods for milk cells and MFG, using RNAlater for stabilization before freezing. Human milk samples (*n* = 26) were collected from lactating participants and either frozen (standard practice) or mixed with RNAlater (1:1, v/v) before freezing. RNA was extracted from separated cellular and MFG fractions and assessed for concentration, quality (RNA quality number-RQN and 28 S/18S ratio), and gene expression (*ACTB*, *LALBA*, *PRLR*, *PTPRC*) using RT-qPCR.

Samples preserved with RNAlater showed significantly improved RNA integrity, particularly in the MFG fraction, compared to those frozen without RNAlater. Gene expression was largely stable across preservation methods. Delays in mixing with RNAlater led to declining RQN values in milk cell fractions, underscoring the need for prompt stabilization. Lastly, we show that this method can be used in low-resource countries by extracting RNA from samples collected in a randomized clinical trial in Guinea-Bissau and shipped to the US for analysis. This procedure led to improved yield and integrity of these samples.

These findings demonstrate that RNAlater pre-freezing stabilization enhances RNA quality and yield, and supports its use for milk gene expression analysis. This approach provides a practical, scalable solution for RNA preservation in clinical and field research, including remote and low-resource settings, as it requires minimal experience and equipment.

**Supplementary Information:**

The online version contains supplementary material available at 10.1007/s10911-026-09601-2.

## Introduction

 RNA is present in all fractions of human milk, including milk cells, which contain cellular RNA from milk-producing epithelial cells (also known as lactocytes) and immune cells [1–4]; the aqueous phase, which contains cell-free RNA and exosomal RNA; and milk fat globules (MFG), which harbor cytoplasmic crescents derived from the lactocytes cells that secrete them [5, 6]. Over the years, various studies have employed different fractions of milk for RNA extraction, depending on their specific research objectives [7–10]. Among these, MFG RNA is the easiest to measure, as this fraction is particularly rich in RNA and provides direct access to the lactocyte transcriptome without requiring cell sorting [5, 8, 11, 12].

In contrast, RNA extracted from milk cells represents a mixed population, predominantly lactocytes, alongside varying proportions of immune cells [1, 3, 4, 13]. Because of this heterogeneity, bulk RNA extraction and sequencing from milk cells can make it challenging to determine the cellular origin of the observed gene expression signals. To address this limitation, some researchers perform cell sorting before analysis [4, 13–16]. However, this approach can be technically demanding and, in some cases, the number of sorted cells may be insufficient for downstream analyses, depending on the sample quality. An alternative strategy involves sequencing the entire milk cell pellet and applying computational deconvolution methods to infer cell proportions in the original sample [10]. These deconvolution algorithms typically rely on reference datasets from single-cell RNA sequencing (scRNA-seq) to predict the cellular composition and disentangle transcriptional signals from complex mixtures. This approach can be particularly useful when sorting is not feasible, offering a computational avenue for exploring the transcriptomic contributions of different cell types in milk samples.

Milk RNA in not stable upon freezing, and even a single freeze and thaw cycle can results to poor RNA quality. In many study sites, especially in low- and middle-income countries (LMICs), involving at-home sample collection, the ability to utilize protocols that require laboratory equipment and trained personnel is often limited. The same is true for most clinical research settings, where such resources may not be readily available, limiting the ability to obtain high-quality RNA from milk samples. To address these challenges, we developed a simplified protocol to preserve human milk samples using RNAlater, a commercially available RNA stabilization reagent (ThermoFisher). This method provides a practical solution for preserving samples in settings with limited access to laboratory infrastructure, enabling researchers to securely store milk for subsequent downstream RNA extraction. Herein, we share results from our analysis demonstrating the efficiency of RNAlater in preserving RNA quality from both MFG and milk cells, highlighting RNAlater’s potential utility for studies conducted in resource-constrained environments.

## Methods

### Participant Cohort

Study population characteristics are listed in Table 1. Milk samples for this study were collected from the UCSF Infant Growth and Milk Supply (IGMS) study, and the samples from Guinea-Bissau were collected as part of the Preventing Infant Malnutrition with Early Supplementation (PRIMES) study [17, 18]. The institutional review board of the University of California, San Francisco, approved the IGMS study (#19-29297) and the PRIMES study was approved by the Guinea-Bissau Comite Nacional de Etica na Saude (Guinea-Bissau National Committee on Ethics and Health, 075/CNES/INASA/2020) and the University of California San Francisco Institutional Review Board (#19-29405). Written informed consent was obtained from all study participants.

**Table 1:** Study population characteristics

Characteristic	IGMS study	PRIMES study
	All Participants (*n* = 15)	All Participants (*n* = 10)
Maternal characteristics		
Age, years, mean (SD)	36 (3.3)	26 (5.71)
Race, n (%)		
African		10
Asian	3	
Caucasian	6	
More than one	2	
Not reported	4	
Ethnicity		
Hispanic, Latino, or Spanish origin	1	
Not of Hispanic, Latino, or Spanish origin	10	10
Not reported	4	
Employment status, n (%)		
No paid employment	1	
Maternity Leave	13	
Sleep & work at home		10
Not reported	1	
Pregnancy and delivery characteristics	(*n* = 12, not reported *n* = 3)	(*n* = 10)
Number of pregnancies, mean (SD)	2 (1.28)	NA
Number of live births, mean (SD)	1.67 (0.89)	1.8 (0.92)

### Sample Collection

In the PRIMES study, samples were collected at the 30-day study visit at home, before feeding. Participants were asked to hand express 10 mL of breast milk from each breast for a total of 20 mL. Samples from both breasts were mixed, placed in a cooler with ice, and transferred to the study site within 6 h after collection. At the study site, 2mL of milk was transferred to a premade and labeled 15 mL tube, containing 2 mL of RNAlater (1:1 ratio). Samples were mixed by inverting the tubes a few times, and were placed in -20 °C until shipping to UCSF on dry ice for analysis. A detailed Standard Operating Procedure (SOP) protocol can be found in Appendix 1.

In the IGMS study, samples were collected at days 7, 14, 21, and 30, as well as at 3 and 6 months postpartum. Participants were instructed to wait 2 h after the last feeding/pumping (range reported 1.5–3 h), sterilize their own pump,wash their hands, and perform full breast expression from both breasts, using a pump at home. If needed, we provided a sterile pump kit (Ameda Dual HygieniKit Milk Collection System) and a pump (Ameda Mya Joy). From each breast, participants were asked to pour a portion of the fresh human milk samples (10–30 mL) into sterile containers. Samples were brought to the lab on ice within 0.5 to 10 h (*n* = 15), or were expressed in the lab lactation room (*n* = 1). If the sample collection was not scheduled immediately after pumping, participants were instructed to refrigerate (4 °C) them or keep them on ice at home until they were brought to the lab. Ten to 30 mL of milk was collected from each breast for analysis in the lab. The time of milk expression reported by the mother and the time of sample processing initiation reported by the lab member processing the sample were documented.

### Sample Processing at the Lab

**Fig. 1:**
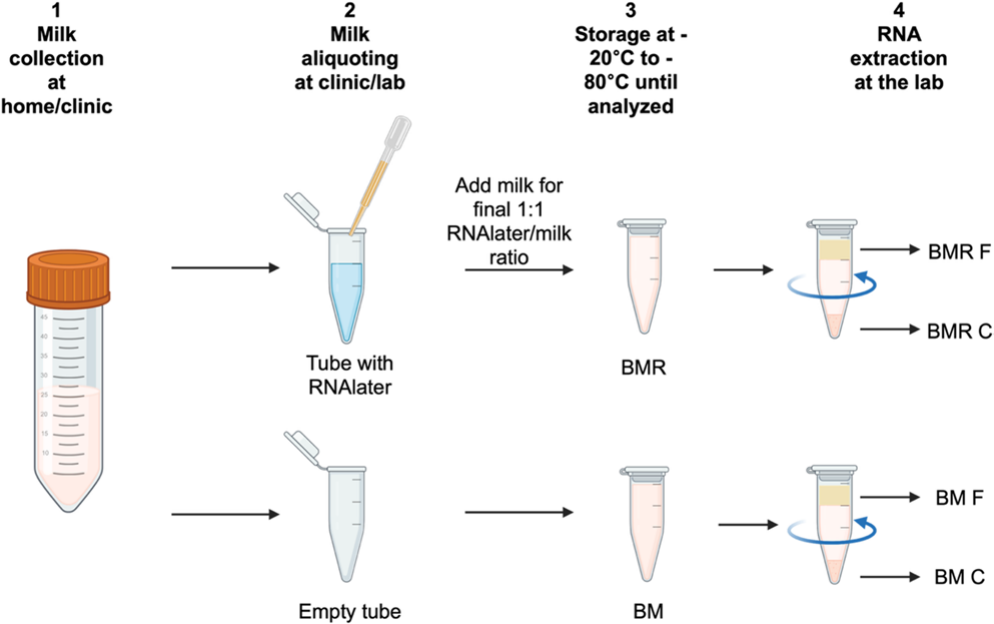
Overview of sample processing. Human milk samples were collected either at the clinic or at participants’ homes. Samples were aliquoted into tubes with or without RNAlater, either at the clinic (PRIMES study) or in the laboratory (IGMS study), and then shipped and stored at the laboratory until RNA extraction. On the day of extraction, samples were thawed, centrifuged, and RNA was extracted separately from the upper milk fat globule (MFG) layer (F) and from the cellular pellet (C)

Each sample received from the IGMS study was aliquoted into 5 or more DNA LoBind tubes (Eppendorf, Catalog # 022431021). In addition, 2 mL of milk was mixed with 2 mL RNAlater (ThermoFisher, Catalog # AM7021), and was aliquoted into two 2 mL DNA LoBind tubes as soon as possible after being received in the lab (only one aliquot of 1 mL milk and 1 mL RNAlater was used for later analysis). Once received and processed at the lab, samples were kept at -80 °C until RNA extraction (samples stored for 256–652 days). For this study, 16 human milk samples from the IGMS study and 10 from the PRIMES study were used; a portion of each sample was mixed with RNAlater (BMR), and a separate portion of each sample was frozen at the same time frame without mixing with RNAlater (BM) (Fig. 1). BM samples from the PRIMES study were thawed once for aliquoting purposes before the RNA extraction.

### RNA Extraction

All RNA extraction steps were performed in the lab. To better understand the effect of mixing samples with RNAlater on RNA measurement in the different milk fractions, we centrifuged each sample after thawing to separate the milk cells (Cellular/C) from the milk fat globules (MFG/F) and extracted RNA from each fraction separately. RNA was extracted from the two fractions (cellular and MFG) under two preservation conditions (with and without RNAlater). A total of 96 RNA samples were extracted and tested in this study: 6 BM and 10 BMR samples from the PRIMES study, and 16 BM and 16 BMR samples from the IGMS study, with each sample providing a cellular and MFG RNA extract. On the day of analysis, BM and BMR aliquots from each sample were thawed on ice (for about 30 min until the samples fully liquified) and centrifuged for 5–10 min, 4 °C at 12,000–15,000 g. The upper layer of MFG (F) was separated into a new tube and mixed with Qiazol lysis reagent (Qiagen, Catalog # 79306). Supernatant was carefully removed using a pipette, and the cell pellet (C) was examined, mixed with Qiazol lysis reagent, and transferred to a new tube. Samples were vortexed to lyse the fat and cells, and RNA was extracted using the RNeasy Plus Universal Mini Kit (Qiagen, Catalog # 73404) according to the manufacturer’s protocol, omitting the TissueLyzer step, which was deemed unnecessary for these types of samples.

### RNA Quality Measurements

RNA concentration was measured using Nanodrop™ One Spectrophotometer (Invitrogen Catalog # 13-400-518). The 5200 Fragment Analyzer system was used to measure RNA Quality Number (RQN) and 28s/18s ratio of RNA samples from the IGMS study, and the 4200 TapeStation system with a high-sensitivity RNA kit was used for samples from the PRIMES study.

### qPCR for Gene Expression

Reverse transcription was performed with 500 ng of total RNA using qScript cDNA Synthesis Kit (Quantabio, 95047) following the manufacturer’s protocol. qRT-PCR was performed on QuantStudio 6 Real Time PCR system (ThermoFisher) using PerfeCTa™ SYBR^®^ Green FastMix™, Low ROX™ (QuantaBio, 95074) with the primers for detection of *ACTB*,* LALBA*,* PRLR*, and *PTPRC* listed in **Table ****S1**. To evaluate whether gene detection/expression differs between the treatments (BMR vs. BM), we used the ΔΔCt method to estimate the fold change in RNA content, with *ACTB* serving as the housekeeping gene. The BMR samples, which were processed with a shorter time interval (0.5 h), served as the control. Fold change = 2^-ΔΔCt^. ΔΔCt = ΔCt (RNA extracted from each sample) - ΔCt (RNA extracted from BMR sample processed after 0.5 h from the same milk fraction). ΔCt (mRNA) = Ct (mRNA of each sample)-Ct (ACTB of the same sample).

### Statistical Analysis

Statistical analysis and figures generation were performed using Prism 10 for macOS (Version 10.4.1). An ordinary one-way ANOVA with Tukey’s multiple comparisons test was used to compare the fractions and treatments for each parameter tested (between-groups comparison). In addition, we performed a paired student’s t-test analysis to look for differences in concentration and fold change expression between the treatments (BMR vs. BM) in each fraction separately. Significance for all statistical analyses was set at *p* ≤ 0.05.

## Results

### RNA Integrity of Human Milk Declines Sharply with Freezing, but can be Preserved by Mixing Milk with RNAlater before Freezing

 Over the decades, human milk samples collected in large cohorts have typically been stored at temperatures ranging from − 20 to -80 °C until analyzed [10, 19]. This traditional freezing process often results in significant degradation of the RNA content in milk, rendering these samples unsuitable for sequencing. To address this issue, we conducted an experiment to assess the ability of RNAlater, an RNA-preserving reagent, to maintain RNA quality and integrity in milk when mixed in a 1:1 ratio before freezing.

**Fig. 2:**
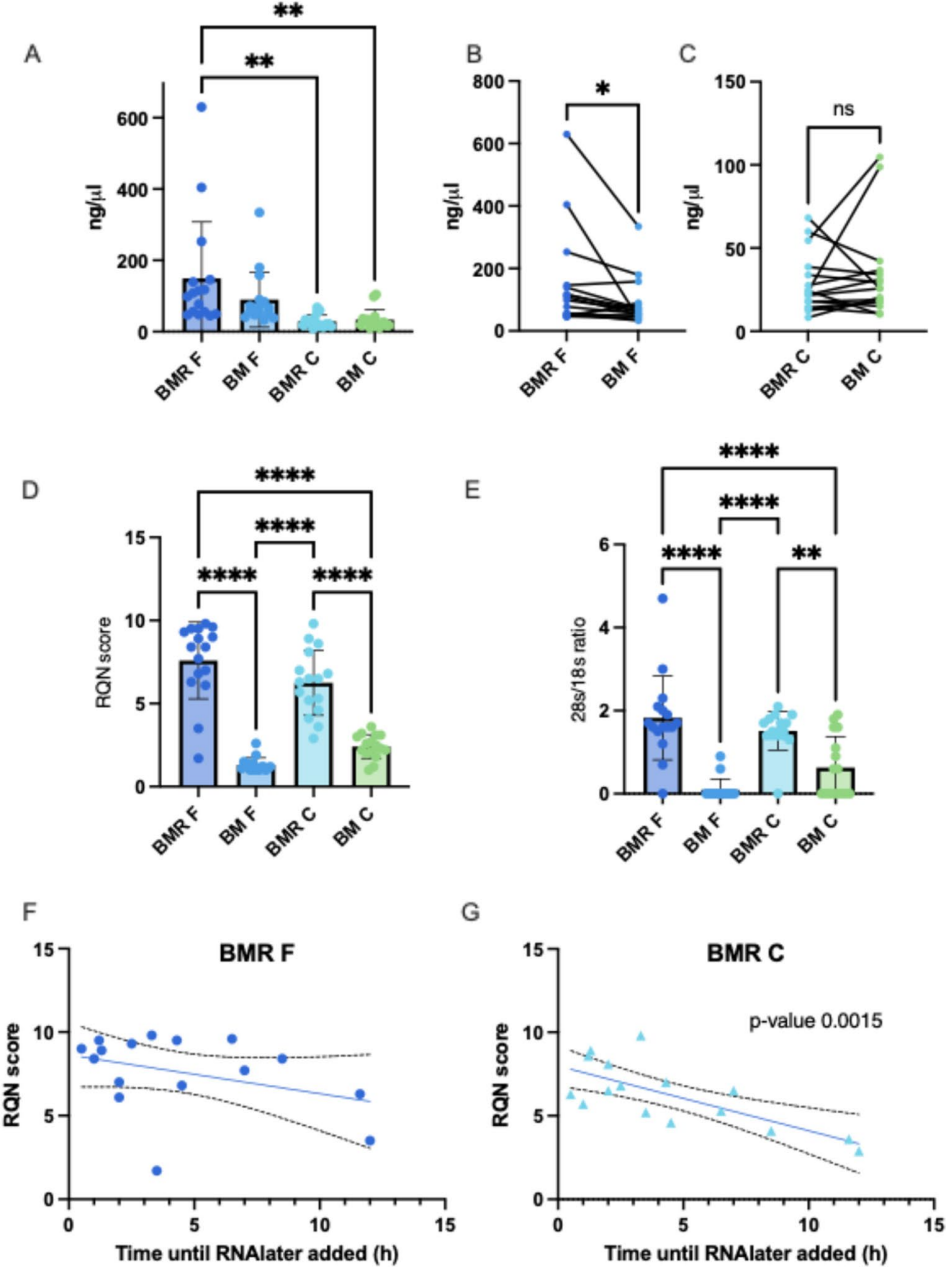
Comparison of RNA quality and yield in human milk samples (*n* = 16) frozen with and without RNAlater. (**A**) RNA concentrations (ng/µL) measured from each fraction are shown on the Y-axis for samples extracted from MFG and milk cells under different conditions (as indicated on the X-axis). Asterisks represent significant differences between groups based on Tukey’s multiple comparison test. (**B**) Paired t-test analysis comparing RNA concentrations from MFG samples preserved with RNAlater (BMR F) versus without RNAlater (BM F), extracted from the same milk sample. (**C**) Paired t-test analysis comparing RNA concentrations from milk cells (**C**) preserved with RNAlater (BMR C) versus without RNAlater (BM C), from the same milk sample. (**D**) RNA Quality Number (RQN) values and (**E**) 28 S/18S ratios of samples from each fraction are displayed on the Y-axis for the conditions indicated on the X-axis. Asterisks represent significant differences between groups using Tukey’s multiple comparison test. (**F** and **G**) Scatter plots depicting how RQN values change when samples are mixed with RNAlater at increasing time points post-milk expression. The Y-axis represents RQN scores, while the X-axis shows the time elapsed (hours) before RNAlater addition. Simple linear regression analysis was performed, and the mean regression line is shown along with the 95% confidence interval (CI). Significant differences are indicated as *p < 0.05, **p < 0.01, ***p < 0.001, ****p < 0.0001

Samples were collected between days 7 to 180 postpartum, and participants’ characteristics are in Table 1. To explore the differences between BM and BMR samples in milk RNA preservation, we examined samples from the IGMS study preserved using a similar approach, with or without RNAlater before freezing (Fig. 1**).** RNA concentration was markedly higher in the MFG fraction than in milk cells across all tested conditions (Fig. 2A). All samples started with either 1 mL of milk combined with 1 mL RNAlater or 1.5-2 mL of milk without RNAlater. When comparing RNA concentrations between the MFG samples preserved with RNAlater (BMR F) and those frozen without RNAlater (BM F), we found higher RNA yields in the BMR F group, on average by 60%, even though they began with a smaller milk volume (Fig. 2B). No significant difference in concentration was found between the cell treatments, with a mean difference of 5 ng/µl (SD = 26.3) (Fig. 2C). These results imply that adding RNAlater before freezing may improve RNA yield in MFG. In addition, our results demonstrated that RNA extracted from both the MFG fraction (BM F) and milk cells (BM C) of frozen samples without RNAlater had substantially lower RNA Quality Number (RQN) scores and 28 S/18S ratios compared to samples mixed with RNAlater before freezing (BMR F and BMR C, respectively) (Fig. 2D and E). RQN scores in all samples frozen without RNAlater were consistently below the recommended levels for RNA sequencing, highlighting significant RNA degradation, and the lower 28 S/18S ratios observed in these samples further confirmed the extent of RNA degradation in the absence of RNAlater stabilization. These results indicate that pre-freezing stabilization with RNAlater significantly improves both RNA quality and integrity in milk samples.

We next aimed to determine the time frame after expression that samples should be mixed with RNAlater to provide data to inform sample collection instructions in a clinical research setting. In MFG, we observed that RQN values remained high when samples were mixed with RNAlater up to two hours after milk expression (Fig. 2F**)**. After this time, some samples showed lower RQN values, although there was no significant correlation between the time elapsed since milk expression and the RQN, suggesting that samples mixed at later time points might still be of good quality for sequencing. In the milk cell fraction, however, a significant reduction in RQN was observed over time since collection, as indicated by a simple linear regression model (Fig. 2G**)**. Despite this, the earliest samples (that were mixed with RNAlater two hours or less after expression) still exhibited relatively lower RQN scores compared to their matched MFG samples (Fig. 2G). This suggests that factors other than time since expression, such as RNase activity in an individual sample, might affect the RNA integrity. Interestingly, milk cell pellets after freezing with RNAlater (BMR C) appeared visibly larger than those from samples frozen without RNAlater (BM C) (Figure S1A-C). Despite the increased pellet size, RNA yield did not rise, likely due to pelleting of additional non-cellular components, such as salts or debris introduced by the high salt content of RNAlater. The purity values (A260/A280 and A260/A230 ratios measured by nanodrop) were comparable between the BM to BMR samples, and were significantly lower in the cell samples (BM C and BMR C) compared to MFG samples (BM F and BMR F)(Figure S1D-E).

### Impact of RNAlater on Gene Expression Patterns Across Milk Fractions

**Fig. 3:**
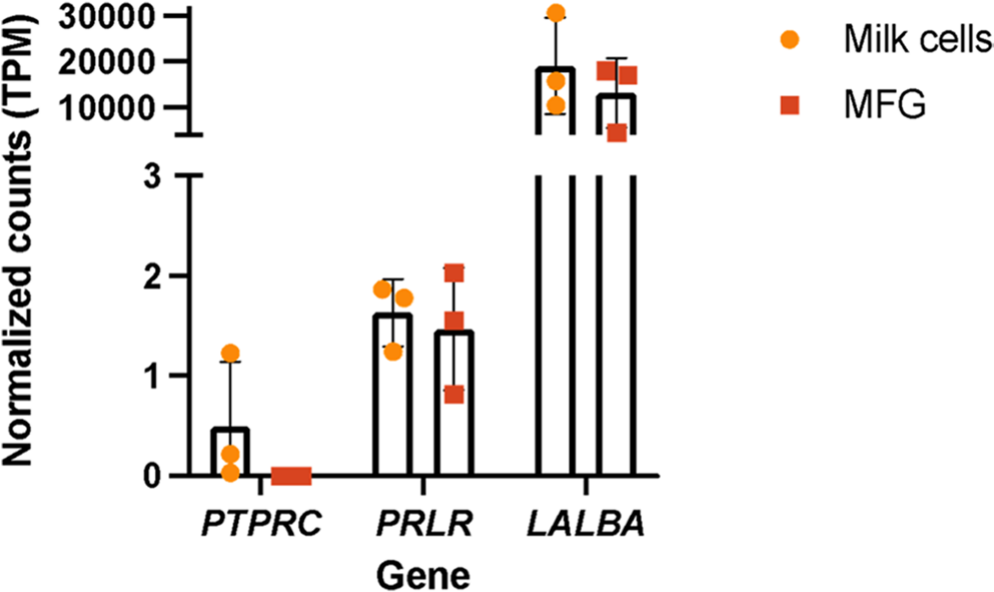
Gene expression profiles from RNA sequencing of milk fat globules (MFG) and milk cells from three fresh milk samples. Read counts were normalized to transcripts per kilobase million (TPM) and are plotted on the Y-axis. Gene names for transcripts (*PRLR*, *LALBA*, and *PTPRC*) are displayed on the X-axis. Bars represent mean TPM values, and error bars denote variability across the three samples

For our qPCR analysis, using *ACTB* (beta-actin) as a housekeeping gene, we targeted three genes with different expression patterns based on our previous RNA-sequencing analysis comparing milk cells and MFG RNA [6]. *PRLR* (prolactin receptor), which is expressed in low levels in MFG and milk cells; *LALBA* (alpha-lactalbumin), which has very high expression in these fractions during lactation; and *PTPRC* (protein tyrosine phosphatase receptor type C, also known as CD45), which is not expressed in MFG and is expressed in milk cells samples with a high portion of immune cells (Fig. 3).

**Fig. 4:**
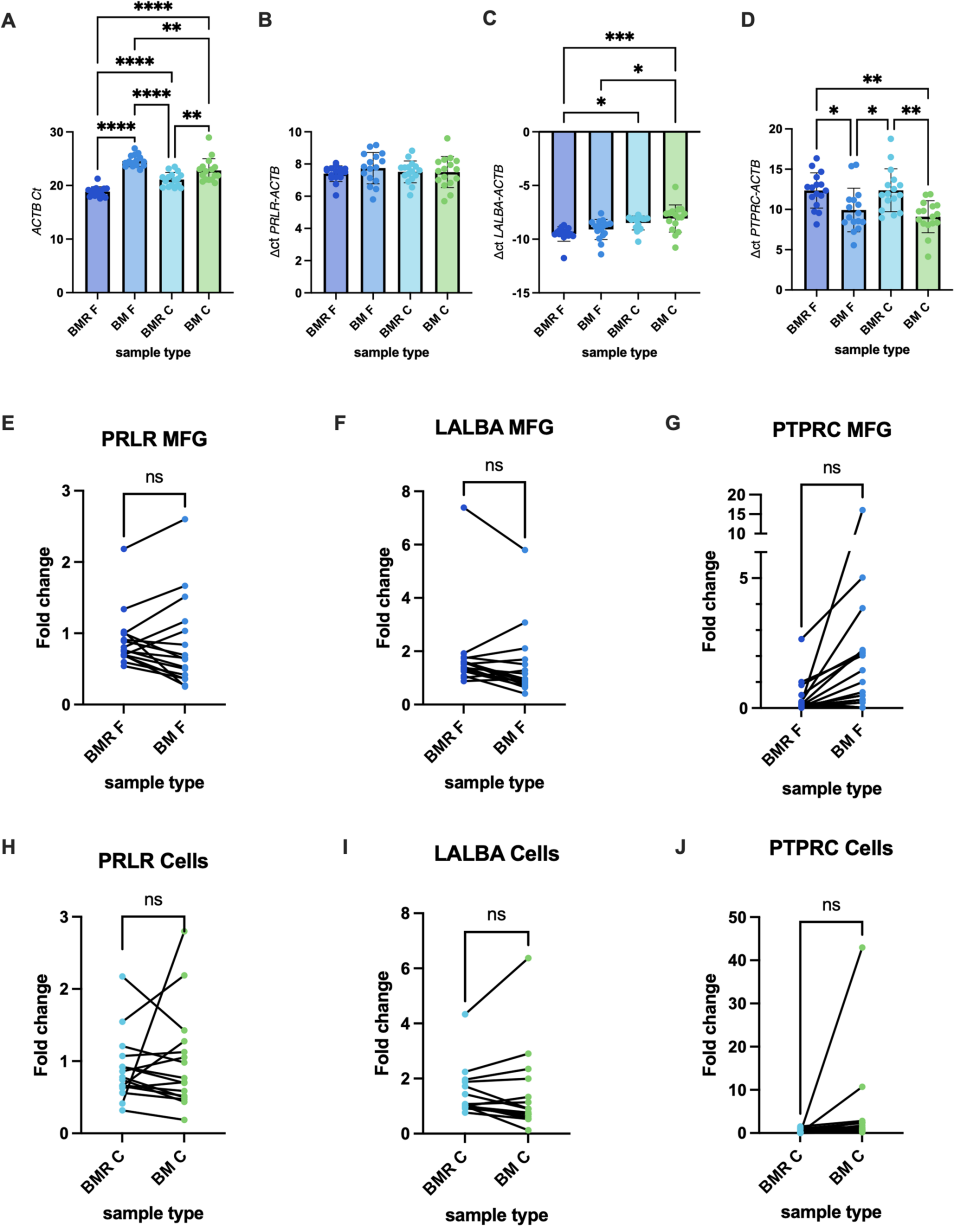
Effect of preservation method on gene expression measured by qPCR. **A** Ct values of the *ACTB* housekeeping gene across different milk fractions (MFG [F] and cell [C]) and preservation methods: BM (standard freezing method) and BMR (with RNAlater). **B**–**D** ΔCt values for *PRLR* (B), *LALBA* (**C**), and *PTPRC* (**D**), calculated as gene Ct – *ACTB* Ct. Lower Ct or ΔCt values indicate higher gene expression. Bars represent the mean ± SEM with individual sample points overlaid. **E**-**G** Fold change in expression of *PRLR* (**E**), *LALBA* (**F**), and *PTPRC* (**G**) genes in the MFG fraction. **H**-**J** Fold change in expression of *PRLR* (**H**), *LALBA* (**I**), and *PTPRC* (**J**) genes in the milk cells fraction. Fold change was calculated relative to expression in samples preserved in RNAlater within 0.5 h post-expression (reference). Statistical comparisons reflect differences between BMR and BM treatments. Lines connect data from the same sample across different milk fractions. Each dot represents the mean value of 2–3 experimental measures from each sample. Significant differences are indicated as *p < 0.05, **p < 0.01, ***p < 0.001, ****p < 0.0001

Using specific primers for these genes, we aimed to evaluate whether low RNA Quality Number (RQN) scores affect the expression patterns of these genes and whether mixing milk with RNAlater impacts their expression or proportions across different milk fractions. We observed significantly higher Ct values for the housekeeping gene *ACTB* in milk samples frozen without RNAlater (BM) compared to those mixed with RNAlater (BMR), suggesting that RNA degradation in the BM samples reduces the detectability of genes (Fig. 4A). To normalize for these differences, we adjusted the expression of target genes to *ACTB* by calculating ΔCt values. For *PRLR*, ΔCt values did not differ between preservation methods (BMR vs. BM) or milk fractions (MFG vs. milk cells) (Fig. 4B). This indicates no impact of RNAlater on this gene’s expression, which is considered very low (Ct values of 25–33). In contrast, ΔCt values for *LALBA* were lower (indicating higher expression) in the MFG fraction compared to milk cells, regardless of the preservation method, and no differences between processing methods (BMR vs. BM) were observed within each fraction (Fig. 4C). For *PTPRC*, ΔCt values were higher in BMR compared to BM, reflecting reduced expression of this immune-related gene when RNAlater was used (Fig. 4D). Surprisingly, *PTPRC* expression was not higher in the milk cell fraction compared to the MFG fraction in either processing group, despite milk cells being known to contain more immune cells (Fig. 4D). Additionally, ΔCt values for *PTPRC* in BMR F were higher than in cells without RNAlater (BM C) but not higher than in cells preserved with RNAlater (BMR C), suggesting that RNAlater may alter the immune cell population or reduce their abundance during freezing. Fold change analysis, derived using the ΔΔCt method (comparing test samples to a reference sample mixed with RNAlater 0.5 h post-collection), revealed no significant differences in expression of any gene (Fig. 4E-J**)**. Furthermore, we found no significant correlation between the expression of *PRLR*, *LALBA*, or *PTPRC* and the time elapsed between milk expression and RNAlater mixing (BMR) or direct freezing (BM). These results suggest that RNAlater improves RNA quality and detectability while subtly impacting immune-cells gene expression, possibly due to changes in immune cell integrity during preservation. The reduction of PTPRC expression after RNAlater treatment should be further investigated in relation to specific cell markers, particularly in studies focuses on specific immune cell populations or immune function.

### Assessing the Use of RNAlater for Preserving RNA from Milk Samples Collected in Low-Resource Settings

**Fig. 5:**
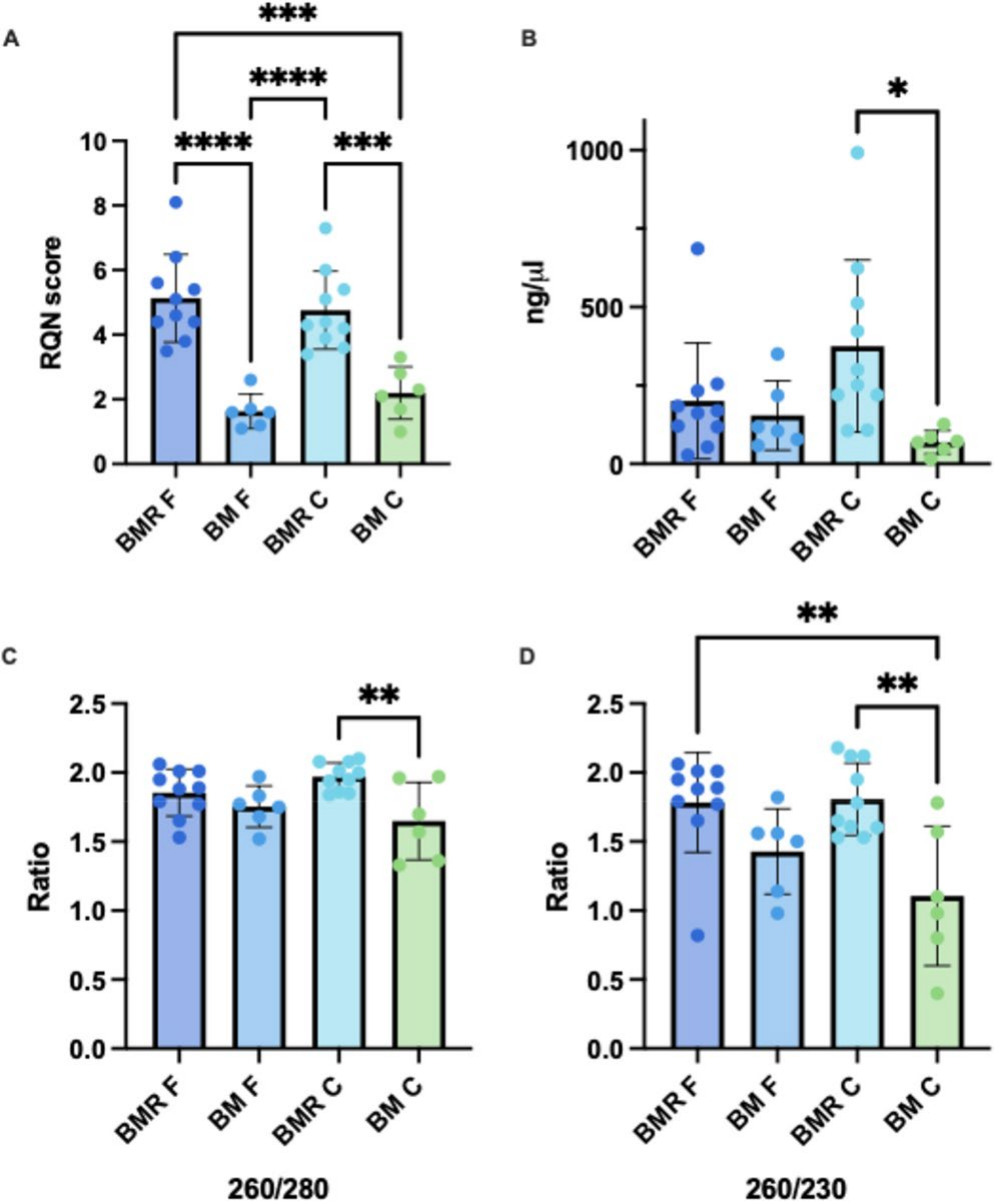
Comparison of RNA quality and yield in human milk samples (*n* = 10) collected at Guinea-Bissau. (**A**) RNA Quality Number (RQN) values are displayed on the Y-axis for samples extracted from MFG and milk cells under different conditions (as indicated on the X-axis). (**B**) RNA concentrations (ng/µL) measured by Nanodrop from each fraction are shown on the Y-axis for. **(C)** RNA purity values A260/A280 and **(D)** A260/A230 ratios measured by nanodrop of samples from each fraction are displayed on the Y-axis. Asterisks represent significant differences between groups based on Tukey’s multiple comparison test. Significant differences are indicated as *p < 0.05, **p < 0.01, ***p < 0.001, ****p < 0.0001

 To evaluate the practicality of using RNAlater for maintaining RNA integrity in milk samples collected under low-resource conditions, we analyzed a subset of 10 samples collected at day 30 postpartum as part of the PRIMES study (Table 1). Samples were collected at participants’ homes, transported to the study site, and mixed with RNAlater before freezing (see Methods, Fig. 1, and Appendix 1 for details). Using this SOP, we observed significantly higher RNA integrity in BMR samples compared to BM samples (Fig. 5A). However, the RQN scores in the BMR F fraction were significantly lower in samples from the PRIMES study compared to those from the IGMS study [mean RQN 5.1 (SD 1.3) vs. 7.5 (SD 2.3), respectively]. No significant differences were observed between the RQN score of BMR C in the studies (when testing all IGMS study time points as a group). RNA concentrations were high and comparable among the BMR F, BM F, and BMR C fractions, whereas BM C samples showed significantly lower concentrations than BMR C (Fig. 5B). The RNA concentration in the BMR C fraction was significantly higher in samples from the PRIMES study compared to those from the IGMS study [mean concentration 375 ng/µL (SD 274 ng/µL) vs. 28.75 ng/µL (SD 17.88 ng/µL), respectively]. RNA purity values were generally within acceptable ranges across all BMR fractions, except for the BM C fraction, which showed reduced purity (Fig. 5C and D).

## Discussion

In this study, we explored methods for preserving RNA quality in human milk samples, specifically focusing on separating the milk fat globules (MFG) and milk cell fractions, utilizing RNAlater as a stabilization reagent for this biospecimen. Our results underline the challenges posed by traditional milk freezing protocols, as samples frozen without RNAlater exhibited substantial RNA degradation, evident by RQN or RIN scores and 28 S/18S ratios below recommended thresholds for RNA sequencing. This highlights the need for stabilization methods that preserve RNA integrity in milk samples, particularly in studies that seek to utilize transcriptomic analyses but do not have the capacity to process fresh milk samples for RNA extraction.

The degradation of RNA upon freezing is often attributed to the inherent presence of RNases and lactoferrin in human milk [20–23]. The use of commercially available preservative reagents was previously shown to be effective in preserving bacterial DNA integrity in human milk samples [24]; however, to our knowledge, the use of preservatives for host RNA stabilization in human milk has not been tested before. Without timely stabilization, freezing alone does not completely inhibit RNase activity, leading to rapid degradation of RNA, as shown by our results and others [10, 25]. Previous studies have sequenced RNA from milk samples stored at -80 °C or samples snap-frozen in liquid nitrogen, despite low RNA integrity scores, and found that milk protein transcripts were the most highly expressed [10, 25], as expected in milk samples. However, using samples with low RNA integrity might affect the expression profile of other genes, and the expression levels observed don’t necessarily reflect their expression at sample collection [26, 27]. It was also previously suggested that cow’s milk has an inherent low RIN score of MFG RNA [25], but our results suggest that this might be a result of the storage methods used, and it is possible that preserving cow’s milk with RNAlater can also improve its RNA integrity.

Here, we also tested this protocol in a subset of samples collected in the field as part of a randomized clinical trial (PRIMES study) in Guinea-Bissau, Africa [17, 18]. RNAlater also improved the quality of samples collected there, although further optimization is needed to improve integrity, such as reducing the time between sample collection and RNAlater addition and improving storage conditions before shipment to the laboratory. The lower RNA integrity observed in BMR samples from the PRIMES study compared to those from the IGMS study may reflect differences in sample handling and storage conditions. Specifically, the reduced RQN scores could be attributed to longer time intervals between collection and freezing, or to temporary storage at − 20 °C before shipment to the United States for analysis, rather than at − 80 °C as performed for IGMS samples. Such factors are known to accelerate RNA degradation, particularly in lipid-rich matrices, where residual RNase activity and temperature fluctuations can rapidly compromise RNA integrity [28]. We also observed higher RNA concentration from the cellular fraction (BMR C) of samples collected in Guinea-Bissau. When comparing results between study sites, it is important to consider that differences in milk expression methods (hand expression versus breast pump) and timing of collection (pre-feeding versus full breast expression) may influence the cellular composition of milk. These biological and procedural differences could also affect RNA concentration and quality in specific milk fractions. Further studies are needed to better characterize how these methodological factors influence RNA yield and integrity, and to develop standardized collection and handling protocols suited for field conditions. Higher RNAlater to milk ratio might help to improve RNA integrity, but this method was not evaluated in this research.

Our analysis of gene expression patterns via qPCR revealed that RNA degradation in samples frozen without RNAlater reduced the detectability of housekeeping genes such as *ACTB*. These findings are in line with previous studies that found that expression patterns might change due to low RIN scores [27]. Normalized gene expression analysis showed that *PRLR* and *LALBA* expression remained unaffected by the preservation method (BMR vs. BM), with *LALBA* exhibiting higher expression in the MFG fraction compared to milk cells. Interestingly, *PTPRC* expression was reduced in RNAlater-preserved samples (BMR C and BMR F), which may reflect changes in immune cell populations during sample freezing and preservation. Contrary to expectations, no higher *PTPRC* expression was observed in milk cells compared to MFG fractions in either processing group, raising questions about how immune-related RNA profiles might shift under different preservation methods. This effect warrants closer investigation, particularly in studies targeting immune cells transcriptomic. The high salt concentration in preservative solution such as RNAlater, may break immune cells membrane, and lead to changes in the cellular pellet of milk. Further studies that uses unbiased techniques such as RNA-sequencing should be perform in the future to address this question. It is notable that fold change analyses and correlations with time until RNAlater mixing did not yield significant differences for any of the tested genes across the 12-hour time interval studied, indicating that immediate stabilization with RNAlater has no adverse effects on gene-specific transcriptional profiles, but is crucial for maintaining RNA integrity overall. Our time-lapse results highlight the importance of timely sample processing, suggesting a two-hour window for RNAlater addition as optimal for preserving RNA integrity in MFG samples. Additionally, they indicate that factors beyond just time, such as RNase activity, sample handling, and others, may contribute to RNA degradation in the milk cell fraction.

In conclusion, stabilizing human milk samples with RNAlater prior to freezing is an effective approach for preserving RNA quality and enhancing RNA yield. Our work underscores the value of RNAlater as a tool for ensuring high-quality RNA recovery from milk fractions, particularly in studies where transcriptomic analyses are integral. Future investigations should examine how RNAlater interacts with specific cell populations and RNA profiles, particularly for immune-related markers, to further refine preservation protocols and optimize their applicability across diverse research settings.

## Supplementary Information

Supplementary Material 1.

Supplementary Material 2.

Supplementary Material 3.

## Data Availability

All data supporting the findings of this study are available within the paper and its Supplementary Information.
