# Supplementary figures and images for: Methods for Preserving Human Milk Cellular and Milk Fat Globule RNA
Source: J Mammary Gland Biol Neoplasia. 2026 Feb 24;31(1):10. doi: 10.1007/s10911-026-09601-2 (PMC13035578; doi:10.1007/s10911-026-09601-2)

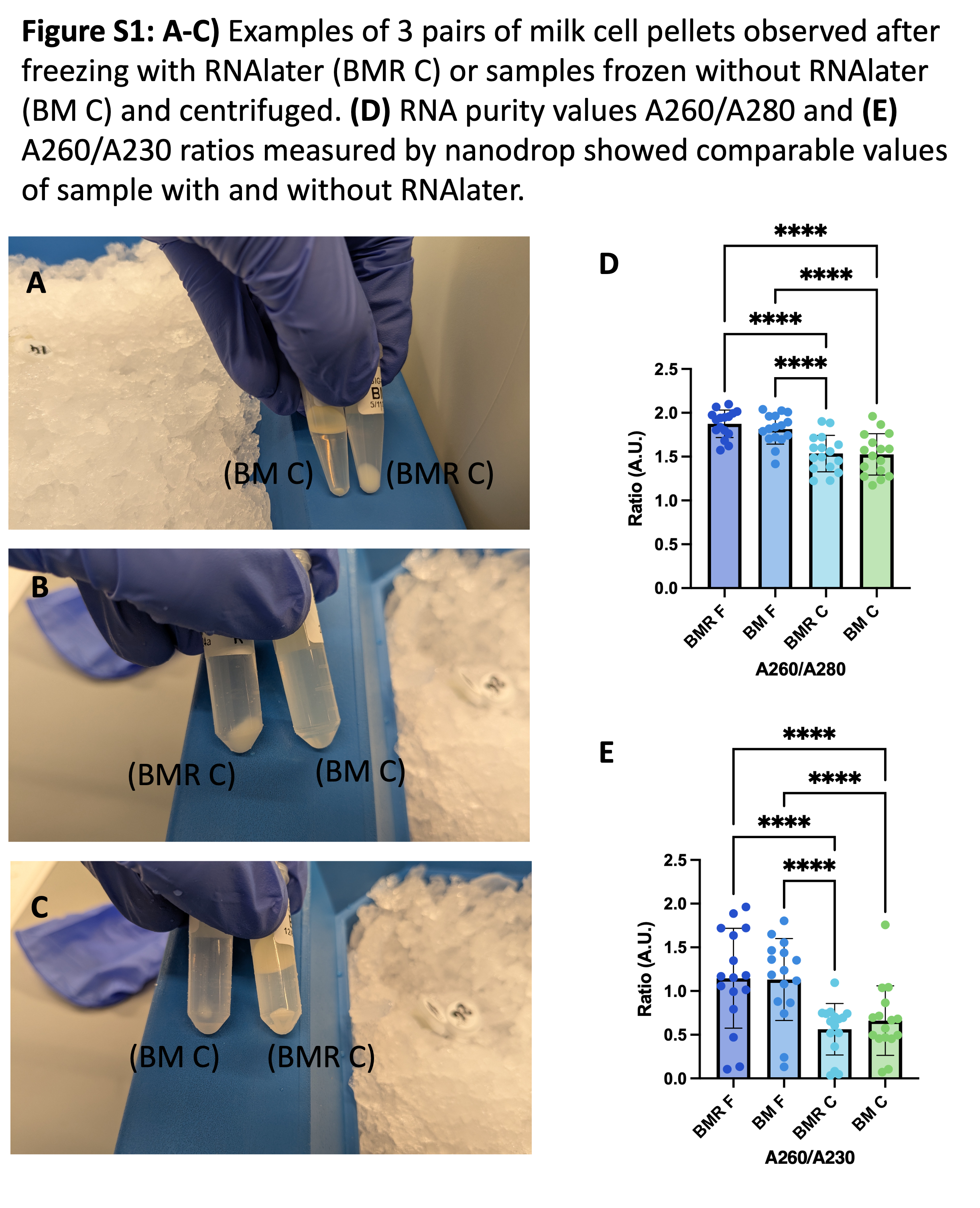

Supplement: Supplementary file 2 — Supplementary Material 2. [file 10911_2026_9601_MOESM2_ESM.png]
