# Supplementary material for: Methods for Preserving Human Milk Cellular and Milk Fat Globule RNA
Source: J Mammary Gland Biol Neoplasia. 2026 Feb 24;31(1):10. doi: 10.1007/s10911-026-09601-2 (PMC13035578; doi:10.1007/s10911-026-09601-2)
Supplement: Supplementary file 3 [file 10911_2026_9601_MOESM3_ESM.odt]

Appendix 1:

Breast Milk Collection Standard Operating Procedures

- Purpose 

This Standard Operating Procedure (SOP) is intended for research staff responsible for collecting study data and biological samples. All personnel involved in sample collection and data handling must complete appropriate research ethics and study-specific procedural training before performing study-related activities. This protocol was developed for the collection of mature milk samples (day 30 postpartum) in a low-resource study site. For milk collected during the early postpartum period or in other settings, additional adaptation may be needed, specifically regarding the timing of collection (before or after feeding), and the volume collected (full breast expression, versus fore or hind milk).

- Materials and Equipment 

1 Breast milk collection containers

1 RNAlater test tube (15 mL canonical tubes filled with 2 mL RNAlater)

Transfer pipette

Biohazard bag 

Cooler 

Icepacks 

Labels 

Water 

Soap

Single-use towel 

- Methods 

- Breast milk samples should be collected from enrolled mothers before breastfeeding and, if possible, after at least two hours have passed since the previous breastfeed.  

- No ointment or topical solution (e.g., lanolin, antibiotics, antiseptics, anti-inflammatories, and homeopathic products) should be applied after breastfeeding immediately before collecting the breastmilk sample.  

- Give the mother water, soap, and a single-use towel to wash their hands immediately before the collection.

- Milk samples must be collected in a clean breastmilk collection container.

- Instruct the mother to place the clean milk collection container underneath the nipple within the mammary areola.

- Instruct the mother to place her fingers about 2-3 cm behind the areola and apply gentle pressure towards the areola to squeeze milk.

  - 10 mL (2 teaspoons) milk from her right breast into the milk container and 10 mL (2 teaspoons) milk from her left breast into the breastmilk container. 

  - Using an unused, clean transfer pipette, transfer 2 mL of milk from the milk collection container into the test tube containing 2 mL RNAlater.

NOTE: RNAlater + breastmilk should reach the 4mL mark on the test tube. It should be a 1:1 ratio in RNAlater.

- If any RNAlater is spilled or leaked, refill (with the provided extra RNAlater solution) the test tube to assure the 1:1 ratio of 2 mL RNAlater and 2mL of breastmilk.

- If a mother is unable to obtain 10 mL from each breast, please collect 20 mL (4 teaspoons) total and mark on the data collection instrument that it was collected disproportionately from the right or left breast.

- Mothers should avoid touching the inside of the milk collection container with their fingers, as well as contact with foreign bodies.

- Do not use intermediate containers (e.g., spoons, bottles, glasses, bottles, etc.) to collect milk before transferring to the sterile container.

- Immediately after collection and aliquoting, close the milk collection container and test tube tightly to avoid spills during transport.

- Label the milk collection container and test tube with Family ID, date, and time of sample collection. 

- Place the labelled specimens into a biohazard bag 

- Place the specimens inside the cooler with frozen ice packs.

Transport the specimens to the sample storage freezer and place in the freezer as soon as possible and within 6 hours at the latest.
